# Anti-Typhoid Inoculation

**Published:** 1904-11-19

**Authors:** 


### The Hospital.

A Journal of

The Medical Sciences and Hospital Administration.

Vol. XXXVII.—No. 947. NOVEMBER 19, 1904.

### Anti-Typhoid Inoculation.

In any country except England, or possibly also Russia, the work which has been done by Professor A. E. Wright, formerly of Netley, and now of St. Mary's Hospital, would be thought to deserve the most cordial assistance from the State and from the military authorities, by reason of the prospect which it affords of preserving armies from the scourge of enteric fever, by which, in so many campaigns, they have been far more seriously crippled than by any injuries inflicted upon them by a human enemy. As a millionaire takes but small account of his halfpence, so it may be assumed that the Russian bureaucracy, resting content upon the resources afforded by a vast population, and profoundly indifferent to the lives or sufferings of individual soldiers, will take no heed whether they live or die. If one Ivan perishes, another must occupy his place. In England a somewhat similar result is brought about by a totally different state of affairs. Behind the War Office there stands the Treasury, and behind the Treasury there stands the election agent, pouring into the ears of the Chancellor of the Exchequer dismal prophecies of what will happen to the "party" if the Budget cannot be kept below a certain amount. In order to meet the election agent's requirements all who point out that, in the conditions of the present day, the country is liable to be plunged into war at brief notice, and that it is our first duty to be prepared in every direction, are at once labelled "alarmists"; and it is assumed that in this way the facts underlying their alarm may be conveniently covered up and put out of sight. It is well known to most householders that very effective reductions of expenditure may often be effected in comparatively small things; and so the War Office, under stress produced by supplies inadequate to its requirements, looks in every direction for opportunities of cheeseparing. It is a natural consequence of the abysmal ignorance of science which usually prevails in high quarters, and especially in high military quarters, that its practical activities in relation to the army are prominent among the matters which are regarded as offering opportunities for the diminution of expenditure, and it is in this way, apparently, that the whole question of anti-typhoid inoculation has been treated. When Professor Wright first introduced it at Netley, he received from his official superiors the sort of liberty which was conferred by the Pickwick Club upon their corresponding members, who, it will be remembered, were left at liberty to pursue their inquiries for any length of time they pleased, upon the condition that they defrayed their own expenses and paid the postages of their letters. Even this degree of freedom was not long permitted; for, upon the recommendation, it was said, of the Advisory Board attached to the Army by Mr. Brodrick, a board partly composed of civilian hospital physicians and surgeons, but not of bacteriologists, and having the supposed function of advising as to the best means of reconciling the reasonable claims of young medical men with the requirements of the service, an order was issued that the inoculations were to be suspended. The question was then referred to two successive scientific committees and both of them recommended that the inoculations should be resumed. Professor Wright, who had originated them, declared that the conditions of complete safety and efficiency had not yet been established, and that his discovery was not ripe for indiscriminate application by comparatively unskilled persons. The War Office, wiser in its own conceit than seven men who could render a reason, directed that the inoculations should be resumed upon the men of a regiment on its way to India, and should be performed by the medical officers who chanced to be in charge. Professor Wright, who until then had been a member of the second scientific committee, at once withdrew from a position in which his advice was disregarded, and has since added materially to his previous very interesting publications on the subject.

It appears from these publications that it is necessary to recognise the existence, as a normal constituent of the blood, of an element, or elements, destructive to pathogenetic bacteria, and that the introduction of such bacteria, in small or moderate dose, produces a sort of rally of the system, during which the destructive elements are elaborated in increased quantity, and the blood becomes more richly stored with them. The completion of the process leaves the subject in a position to resist a larger bacterial dose than he could have resisted in the first instance, and, by a repetition of the process for a sufficient number of times and at sufficient intervals, he may be rendered wholly and, perhaps, permanently immune. On the other hand, the period immediately following an inoculation, either the first or a subsequent one, is liable to be a period of increased proclivity to infection, or, at least, of diminished resistance. Accidental infection in such conditions would be a reinforcement, so to speak, of the designed infection, and the two together might easily be in excess of the resisting powers of the patient. It follows that some test of his condition is required, and that successive inoculations cannot be safely guided by any rule of thumb either as to dose or interval. The test is afforded by an examination of the blood, analogous to that employed for ascertaining the degree of immunity against the bacillus of tubercle which has in the same way been afforded by inoculations, and it is one which requires for its accurate conduct a considerable amount of laboratory experience and of manipulative skill. It is reasonable to hope, of course, that the results of a sufficient number of such examinations may ultimately admit of expression in the form of practical rules, or may be connected with symptoms more easy of observation; and, in that case, the practice of anti-typhoid inoculation would fall into its right position as one of the ordinary resources of preventive medicine. At present, it cannot be said to have attained this position, and it is deeply to be regretted that it has been suffered to pass out of the capable hands of its originator. It is probable that no trephine in ordinary use would make an opening large enough to admit any adequate conception of a scientific question into the brain of what is collectively called the War Office; but it is none the less to be deplored that the attainment of immunity from typhoid in the army should, apparently, be in danger of being indefinitely delayed. No one acquainted with the history of its ravages can regard such a prospect without consternation.

